# High prevalence of hypohydration in occupations with heat stress—Perspectives for performance in combined cognitive and motor tasks

**DOI:** 10.1371/journal.pone.0205321

**Published:** 2018-10-24

**Authors:** Jacob F. Piil, Jesper Lundbye-Jensen, Lasse Christiansen, Leonidas Ioannou, Lydia Tsoutsoubi, Constantinos N. Dallas, Konstantinos Mantzios, Andreas D. Flouris, Lars Nybo

**Affiliations:** 1 Department of Nutrition, Exercise and Sports, Section for Integrative Physiology, University of Copenhagen, Copenhagen, Denmark; 2 FAME Laboratory, Department of Exercise Science, University of Thessaly, Karies, Trikala, Greece; Nottingham Trent University, UNITED KINGDOM

## Abstract

**Purpose:**

To evaluate the prevalence of dehydration in occupational settings and contextualize findings to effects on performance in cognitively dominated tasks, simple and complex motor tasks during moderate and high heat stress.

**Methods:**

The study included an occupational part with hydration assessed in five industries across Europe with urine samples collected from 139 workers and analyzed for urine specific gravity. In addition, laboratory experiments included eight male participants completing mild-intensity exercise once with full fluid replacement to maintain euhydration, and once with restricted water intake until the dehydration level corresponded to 2% bodyweight deficit. Following familiarization, euhydration and dehydration sessions were completed on separate days in random order (cross-over design) with assessment of simple motor (target pinch), complex motor (visuo-motor tracking), cognitive (math addition) and combined motor-cognitive (math and pinch) performance at baseline, at 1°C (MOD) and 2°C (HYPER) delta increase in body core temperature.

**Results:**

The field studies revealed that 70% of all workers had urine specific gravity values ≥1.020 corresponding to the urine specific gravity (1.020±0.001) at the end of the laboratory dehydration session. At this hydration level, HYPER was associated with reductions in simple motor task performance by 4±1%, math task by 4±1%, math and pinch by 9±3% and visuo-motor tracking by 16±4% (*all P<0*.*05* compared to baseline), whereas no significant changes were observed when the heat stress was MOD (*P*>0.05). In the euhydration session, HYPER reduced complex (tracking) motor performance by 10±3% and simple pinch by 3±1% (*both P<0*.*05*, compared to baseline), while performance in the two cognitively dominated tasks were unaffected when dehydration was prevented (*P>0*.*05*).

**Conclusion:**

Dehydration at levels commonly observed across a range of occupational settings with environmental heat stress aggravates the impact of hyperthermia on performance in tasks relying on combinations of cognitive function and motor response accuracy.

## Introduction

The importance of preventing dehydration has received much attention in health and work-safety advisories, from sport coaches, highlighted during hot periods in medias, emphasized in commercial campaigns and recognized by the physiological society as a very important strategy for minimizing the detrimental effects of heat stress on physical performance [1,2]. Nevertheless, dehydration (corresponding to a ~2% loss in body weight or a urine specific gravity [USG] of 1.020 or above) is reported as frequently occurring in occupational settings dominated by many manual, physically demanding tasks and especially so at work-places with high environmental temperatures [3–6]. Clearly, severe dehydration may disturb mood, provoke dizziness, cause confusion and in severe cases lead to delirium, coma and eventually death [7–9]. Dehydration may also provoke fatigue during strenuous physical activity [10–14], especially during exercise in the heat as even mild dehydration impedes thermoregulation, aggravates hyperthermia and compromises cardiovascular function [11,15–22]. Dehydration also accelerates the decline in cerebral blood flow during exercise in heat stress conditions, but the cerebral metabolic rate for oxygen does not decline [19,23,24]. It remains unclear how cognitive functions are affected as long as severe dehydration is prevented i.e. if cognitive functions can be maintained when hydration levels are in the range, which is relevant for working or recreationally active people.

Relating to the effects of dehydration on performance in cognitive or functional tasks, not directly depending on the exercise capacity of the participants, the discrepancies observed between studies may relate to the task complexity, test duration, the magnitude of heat stress, and combination of test applied [25]. We recently observed that hyperthermia (a 2–2½ °C elevation in body core temperature without dehydration) impaired complex motor task performance evaluated with visuo-motor tracking, while there was no influence on the ability to perform a simple motor task (involving pinch to a prescribed force level) and also unchanged performance in a predominantly cognitive task (math addition) [26]. Therefore the complexity within the employed test battery could partly explain the conflicting result reported from laboratory studies [8,27–32]. Secondly, the experimental designs employed so far have not allowed assessment of the separate and combined impact of dehydration and heat stress on cognitive and motor performance. On one hand, Watson and colleagues [33] observed that mild dehydration (~1% water deficit) increased the frequency of errors during a monotonous driving task and Lindseth et al. [34] reported poorer performance for simulated flights in dehydrated pilots compared to a hydrated state. Furthermore, Gopinathan and colleagues [35] evaluated mental performance with word recognition, serial addition and trial-marking tests and reported lower performance when the participants’ dehydration levels was equal to or exceeded 2% in body weight loss. On the other hand, no effects on cognitive performance (executive function, choice reaction, vigilance etc.) have been reported across a series of studies [7,8,31,32,36–39], although the dehydration-levels were comparable to the above-mentioned studies and the participants’ mood and wellness were negatively affected when participants were dehydrated [7,10,31].

The present study was conducted to identify the influence of dehydration on performance in simple and complex motor tasks as well as cognitive dominated and combined motor-cognitive tasks under controlled (laboratory) conditions with and without hyperthermia. The study also incorporates a real-world perspective on the laboratory-based results by including data on the prevalence of dehydration across different occupations with moderate to high heat stress. The latter included an occupational study part with measures of wet-bulb-globe-temperatures (WBGT; as index of the environmental heat stress) and assessment of workers USG levels at the onset and end of work shifts in five different industries representing both indoor and outdoor occupations, including low to high physical activity levels and the ability to utilize motor and cognitive function in both simple and complex tasks, of importance for daily-day performance. The laboratory part was designed to mimic occupational setting with alternating tasks and with a protocol allowing for identifying the influence of dehydration during moderate and high heat stress conditions on 1) simple motor task performance, 2) a task predominantly relying on cognitive function, 3) performance in a combined motor-cognitive task and 4) complex motor task performance.

We hypothesized that dehydration i.e. two percent body weight deficit, would aggravate the impact of heat stress on motor-cognitive test performance and that the complex motor and combined motor-cognitive tasks, would be more susceptible to dehydration and hyperthermia compared to performance in a simple motor or simple cognitive task. The occupational study was observational, with the purpose of providing much-needed data on the hydration status among workers in real-world occupational settings, across different sectors in industrialized countries and assess how hydration levels are affected during a work-shift. The occupations were chosen based on their geographical location, as workers who have these occupations reports that they are suffering from severe heat stress during a work shift. However, we hypothesized that dehydration could be an issue in some occupations, but with lower prevalence compared to previous reports as the awareness of preventing dehydration has received much attention in recent years.

## Methods

### Participants

One-hundred-thirty-nine workers i.e. manufacturing workers (indoor aluminum extrusion; n = 36), agricultural workers (n = 15), police officers (n = 50), tourism workers (n = 26) and construction workers (n = 12), from four different countries (Denmark, Cyprus, Greece and Spain, respectively) participated in the hydration (USG) occupational study and eight able-bodied recreationally active males (Age; 30±2 years, body weight; 85±4 kg) participated in the laboratory part. All participants, received written and oral information of experimental procedures and any risks and discomfort associated with the experiment before they provided written consent to participate in this study, approved by the National Committee on Health Research Ethics *(protocol number*: *55907_v3_02012017)*.

### Experimental design

All participants were unaware of the researchers’ hypotheses and naive to the purpose of the studies. Based on the experimental objectives and hypotheses, it was not possible to blind participants, to the occupational or laboratory experimental conditions involving euhydration and dehydration in combination with moderate and severe hyperthermia.

#### Occupational study

Of the one-hundred-thirty-nine participants, from the four different countries across Europe, one-hundred-nineteen participants provided a urine sample at the ONSET and END of their work shift on the experimental day, whereas twenty workers (manufacturing, Denmark) provided one urine samples at the beginning of their works shift, on the experimental day. Onsite climate measures of air temperature (T_a_), humidity in percentage (%RH) and wet-blub-globe-temperature (WBGT) were obtained with a portable WBGT-103 heat stroke checker (KEM, Kyoto electronic manufacturing CO., LTD., Japan), on the experimental day at the different work places. Climate data was on average: Danish aluminum manufacturing (T_a_; 29°C, %RH; 25 and WBGT; 20°C), Cyprus agriculture (T_a_; 29°C, %RH; 55 and WBGT; 27°C), Greece police officers (T_a_; 27°C, %RH; 50 and WBGT; 24°C), Greece tourism (T_a_; 30°C, %RH; 55 and WBGT; 25°C) and Spanish construction (T_a_; 26°C, %RH; 54 and WBGT; 23°C).

#### Laboratory study

The laboratory study involved familiarization to heating and to the motor and cognitive tasks involved in the study, followed by two different experimental sessions on separate days and conducted in randomized counterbalanced order.

One experimental session involved euhydration (EUH), the other dehydration (DEH). During both of these sessions, the participants completed a test battery consisting of four different tasks at BASELINE, following moderate elevation of core temperature (MOD) and following hyperthermia (HYPER). All testing procedures were conducted in an environmental chamber (University of Copenhagen, DK), at room temperature/air temperature (T_a_); 40°C, relative humid (%RH); 25% and wet bulb globe temperature (WBGT); 29°C. The participants were randomly allocated to either start with EUH or DEH session and then crossover to the other intervention on the subsequent experimental day.

#### Experimental procedure of the laboratory study

Before initiating the experimental sessions, each participant was carefully familiarized with all the included tasks to avoid significant learning effects during the testing sessions, potentially obscuring the behavioral outcome measures. Analogous to the procedure of Piil et al 2017 [26], the participant completed a familiarization heating session in the laboratory, at a room temperature of 40°C to become familiar with heat exposure and the experimental procedures. The familiarization to the battery of motor-cognitive tasks consisted of 2 days with 250 pre-trials of the complex motor task each day and familiarization verified by no improvement in performance during the last 100 of the 500 VMT trials. Familiarization to the remaining tasks were limited to 10 pre-trials as pilot testing demonstrated that participants had no increase in performance after ~5 pre-trials.

For each participant, a minimum of three days and no more than two weeks separated the experimental sessions and all testing including familiarization was completed within one month. In all experimental sessions, the participants completed motor-cognitive tests, at (BASELINE) followed by ~30 min. of ergometer cycling (100 W, ~75 rpm), to increase sweat rate i.e. ensuring evaporative weight loss and to maintain steady core temperature (T_core_), at ~1°C above BASELINE i.e. moderate elevation of core temperature (MOD). Following this, the participants were seated in a conventional office chair, either waiting passively until body weight decreased to 2 percent below BASELINE (DEH) or remained seated in front of the computer and were required to drink in order to match sweat loss (EUH), before completing the motor and cognitive tests again. After finishing the motor-cognitive test battery in MOD condition, the participants returned to the ergometer cycle, for an additional cycling period ~30 min. (100 W, ~75 rpm) until reaching a core temperature >2°C above BASELINE corresponding to a rate of perceived exhaustion (RPE) of ~18 on BORG-scale (RPE ranging—from 9–15 during MOD and from 15–19 during HYPER, across sessions), before repeating the motor-cognitive test battery a third time in HYPER condition. Pilot testing, with 4 hours of consecutive testing (test battery completed every hour) revealed that performance in all applied tasks remained stable, when the hydration (EUH or DEH) was unchanged over time or the heat stress was maintained at MOD levels—signifying that the duration of exposure or the protocol per see did not induce fatigue or changes in performance when conditions remained constant.

All participants arrived at the lab ~30 min. before the start of the experiment. Participants then emptied their bladder into a sealed urine container subsequently analyzed for USG. All participants completed questionnaires reporting thermal comfort rating (TC), temperature sensation rating (TS) as well as modified rating of perceived exertion (RPE; BORG-scale) corresponding to the level of heat stress fatigue and were weighed (without clothes), before a resting heart rate measure was obtained prior to entering the climatic chamber. On the experimental days, in both the EUH and DEH condition, participants were seated for 15 min. in an office chair and completed 40 further familiarization trials of the motor-cognitive test battery, before they initiated the measurements, this to avoid warm-up decrements and to minimize influence on performance during BASELINE measurements [40]. Following completion of the BASELINE test, the participant exercised on an ergometer bicycle (Monark Ergomedic, E839) for ~30 min. at a fixed load (100 W corresponding to a metabolic heat production of ~400 W) with a steady cadence ~75 rpm. During the EUH session, participants were allowed to ingest water with a temperature of ~37°C, when acute thirst response was evoked (with a minimum required amount estimated based on the familiarization session) to prevent dehydration, without directly influencing the core temperature response. During the DEH session, participants did not ingest any liquids until they reached the target (2%) body weight deficit, thereafter participants was allowed to ingest water to keep their body weight deficit steady below BASELINE. Following 30 min. of exercise, the participants returned to the office chair and remained seated for ~5 min., before commencing the motor-cognitive test battery. During both EUH and DEH, participants wore rain clothing to ensure that the core temperature remained stable, at ~1°C (MOD) and ~2°C (HYPER) above BASELINE, respectively.

#### Tests of motor and cognitive performance

**Fig 1:**
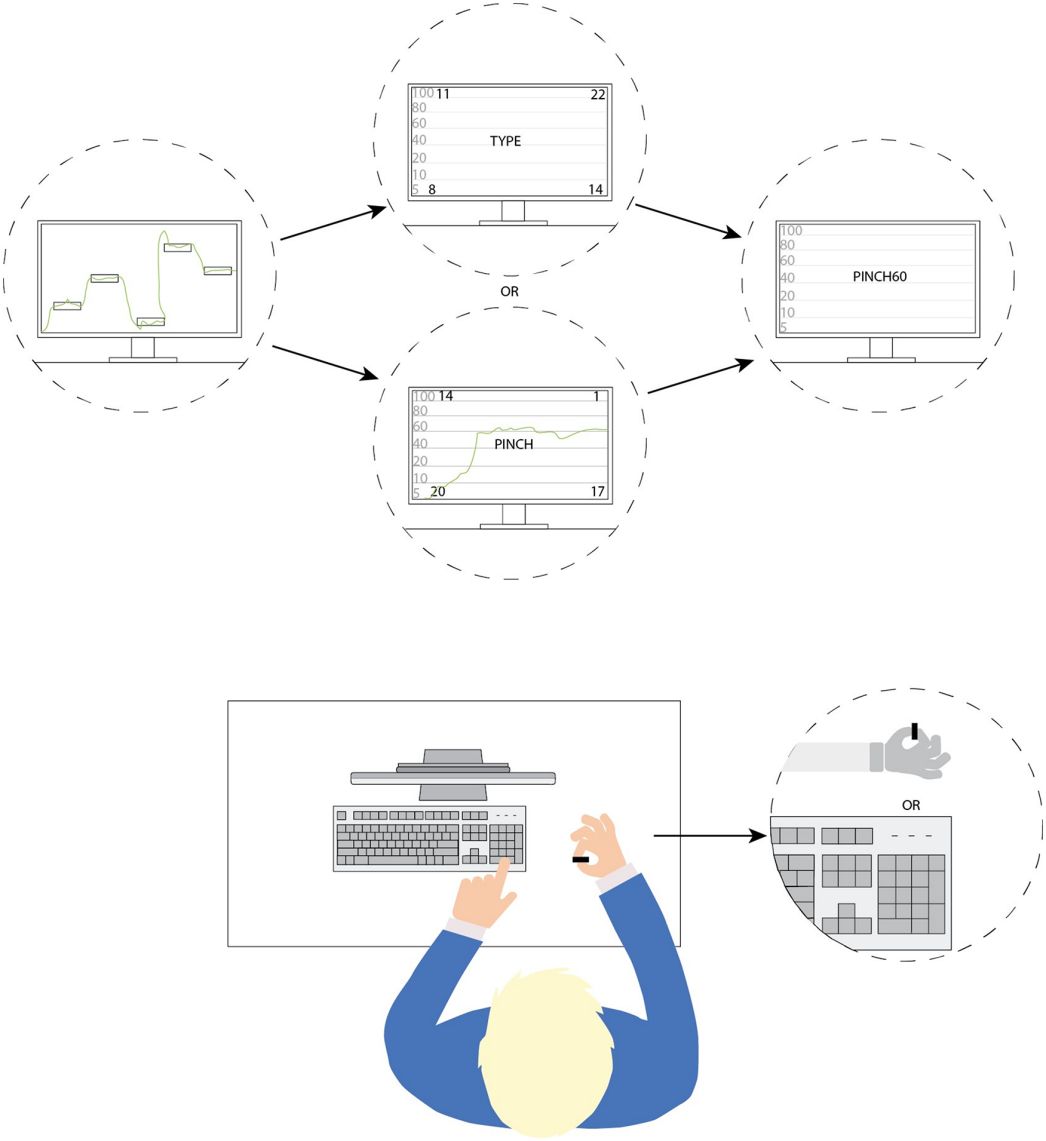
Illustration of the motor and cognitive tests employed in the laboratory study. First task illustrates the visuo-motor tracking task (VMT), where the 5 boxes/targets appears on the screen 1 s before the 12 s tracking begins. The cursor (green line) moves from left to right over the screen (fixed velocity) and the subject is instructed to adjust force on the strain gauge transducer to keep the cursor within the designated box at the given time. VMT alternates with the MATH_TYPE or MATH_PINCH and TARGET_PINCH tasks (listed in the order of how the tasks appear). During VMT, MATH_PINCH and TARGET_PINCH, subjects used the strain gauge, whereas in the MATH_TYPE the number pad was used (bottom pic, right). Figure adopted from [26] with permission.

The motor and cognitive test battery applied in the present study is adapted from Piil and colleagues [26] (see Fig 1).

During all motor-cognitive tests, participants were seated on an office chair in front of a 24-inch computer monitor (Samsung; South Korea). The participant used the preferred (dominant) hand for all tests (all subjects used their right-hand). The motor tasks involved recording of pinch force. Force was applied to a load cell (Dacell, model UU3-K5, 5 Kgf), connected to an amplifier (Dacell, DN-AM-310) and the signal was subsequently digitalized and sampled at 500 Hz, with a data acquisition board, NI-USB-6008 (National instruments inc., USA). A customized script built on PYTHON (Python software foundation, USA) was used for running the motor-cognitive test.

The motor-cognitive tests involved four different task: 1) TARGET_PINCH—a simple motor task: one number (ranging from 1–100) was displayed in the middle of the screen. The number referred to the pinch target force that the participant was required to generate, with the target number changing from one to the next trial. 2) MATH_TYPE—a simple predominantly cognitive task: the sum of four numbers was to be calculated and the result provided by the participant using the numerical keyboard. 3) MATH_PINCH—combined motor-cognitive task: The task was similar to MATH_TYPE but the participants were required to provide the calculated result as a motor response i.e. as a steady pinch force within 6 s and hold the cursor steady, for the remaining 6 s of the task. For both math tasks, the four numbers (ranging from 1–25), were displayed in each corner of the monitor. The participants were then required to add the numbers together and either type the result or pinch the result (adjust force via the load cell, using thumb and index finger). 4) Visuo-motor tracking task (VMT)–complex motor task: the participants were instructed to apply pinch force on a strain gauge to adjust the vertical position of a cursor moving with constant speed across the monitor, this to as accurately as possible hit and stay within five targets (boxes) with different positions on the y-axis (see Fig 1). For every condition (BASELINE, MOD and HYPER) during both session (EUH and DEH), the battery of test lasted 30 min. During the 30 min., a series of 3 tasks, always starting with the VMT tasks followed by either the MATH_TYPE or MATH_PINCH task and every sequence finish with a TARGET_PINCH task. Each tasks lasted 12 s and following each task, visual feedback (1 s) on performance was provided as a score appearing in the bottom right corner of the screen (to encourage the participant to perform as well as possible) and 2 s of transition time to next task, equal 45 s of 40 series (40 sequences of VMT, 40 sequences in total of MATH_TYPE and MATH_PINCH and 40 sequences of TARGET_PINCH). The 30 min. of motor-cognitive tests was chosen to mimic real-working settings with 30–45 min. of continuous work before a period of rest, but also to ensure that any decrements due to fatigue of time would be avoided, as it was observed from pilot studies and familiarization that the motor-cognitive test battery could be maintained for 100 or even 200 consecutive tasks corresponding to ~1 hour of testing in the heat.

#### Thermometry and hydration

T_core_ was measured with a rectal thermometer (Ellab Copenhagen, CTD85) inserted 7–10 cm beyond the anal sphincter. Urine specific gravity (USG) was measured before and after completing the EUH and DEH session, using a refractometer (ATAGO, Tokyo, Japan, pocket refractometer, s/no P811580), to ensure that all participants were hydrated (USG<1.020) before starting the trials and in combination with BODY WEIGHT assessment to track changes in hydration over time in the respective trials. In the occupational setting participants were instructed to deliver one urine sample in the morning during mid-day, in the afternoon and/or in the night, corresponding to when the working hours started and/or finished. Body weight was assessed using a platform scale with accuracy down to 0.1 kg (InBody 270, InBody CO Ltd).

#### Thermal comfort, sensation and RPE

All participants completed questionnaires before, midway and after the motor-cognitive test-battery. The questionnaires were standardized (ASHRAE standard 55), for thermal comfort rating (TC), temperature sensation rating (TS) and BORG-scale (rate perceived exhaustion—RPE) corresponding to the heat stress level of fatigue.

### Data analysis

For all the four motor-cognitive tests, the best possible performance score was 100 percent. In the TARGET_PINCH, MATH_TYPE and MATH_PINCH tasks, the score is the percentage of the correct answers provided. In the MATH_TYPE task, it was possible to provide a 100% correct answer as the calculated result was typed using the numerical keyboard—therefore it was also possible to distinguish between a correct/un-correct answers. In the MATH_PINCH it was more difficult to distinguish between correct/un-correct answer, due to the oscillations when pinch force was applied to the load cell. Therefore, we only calculated the correct/un-correct answers for the MATH_TYPE task. For the VMT task the score was the percentages time on target i.e. 100 percent equals maximum score, if the cursor was keep inside the five boxes for the entire time.

### Statistical analysis

Comparison of all values from tests (TARGET_PINCH, MATH_TYPE, MATH_TYPE_ERRORS, MATH_PINCH and VMT) and for T_core_, body weight, USG, HR, TC, TS and RPE) were analyzed using linear mixed models with group-time interactions (hydration and temperature) as fixed effects and participants as random effects, these were included in the models to account for the dependency between measurements on the same subject. All tests were carried out with the statistical program R (R Core Team, 2015) with a linear mixed model approach applied to the data incorporating the functionality of the package *lme4* [41], while the specific comparisons and the corresponding t-tests and adjusted p-values were calculated using the package *multcomp* [42]. Incorporating these models into R makes it possible to compute specific comparisons (with parameterization as specified by the formulas below), thereby allowing the comparisons of importance for the present study.

Parameter specification for the linear mixed models incorporated to R:

$$\begin{array}{ll} p1.m3<-glht(m3,linfct & \\ & =c("HydraTempEUH.HYPER-HydraTempEUH.BASELINE=0",\\ & "HydraTempEUH.MOD-HydraTempEUH.BASELINE=0",\\ & "HydraTempEUH.HYPER-HydraTempEUH.MOD=0")) \end{array}$$

$$\begin{array}{ll} p3.m3<-glht(m3,linfct & \\ & =c("HydraTempEUH.HYPER-HydraTempEUH.BASELINE\\ & -HydraTempDEH.HYPER+HydraTempDEH.BASELINE=0",\\ & "HydraTempEUH.MOD-HydraTempEUH.BASELINE\\ & -HydraTempDEH.MOD+HydraTempDEH.BASELINE=0",\\ & "HydraTempEUH.HYPER-HydraTempEUH.MOD\\ & -HydraTempDEH.HYPER+HydraTempDEH.MOD=0")) \end{array}$$

Model validation included visual inspection of residual plots and normal probability plots. To accommodate the specific hypotheses for the study, specific sets of contrasts comparing within-session-changes (BASELINE, MOD and HYPER) and between the two sessions (EUH and DEH). Specifically, approximate global F-tests were carried out and, subsequently, model-based t-tests were used to identify the significant differences.

The resulting p-values were obtained from a standard normal distribution and multiplicity adjusted using the single-step method, a recently developed procedure providing a less conservative adjustment of p-values as compared to Bonferroni, adjustment and related adjustments in order to control inflation of family-wise type I error rate, by utilizing the correlations between tests.

## Results

### Occupational study

#### Hydration

**Fig 2:**
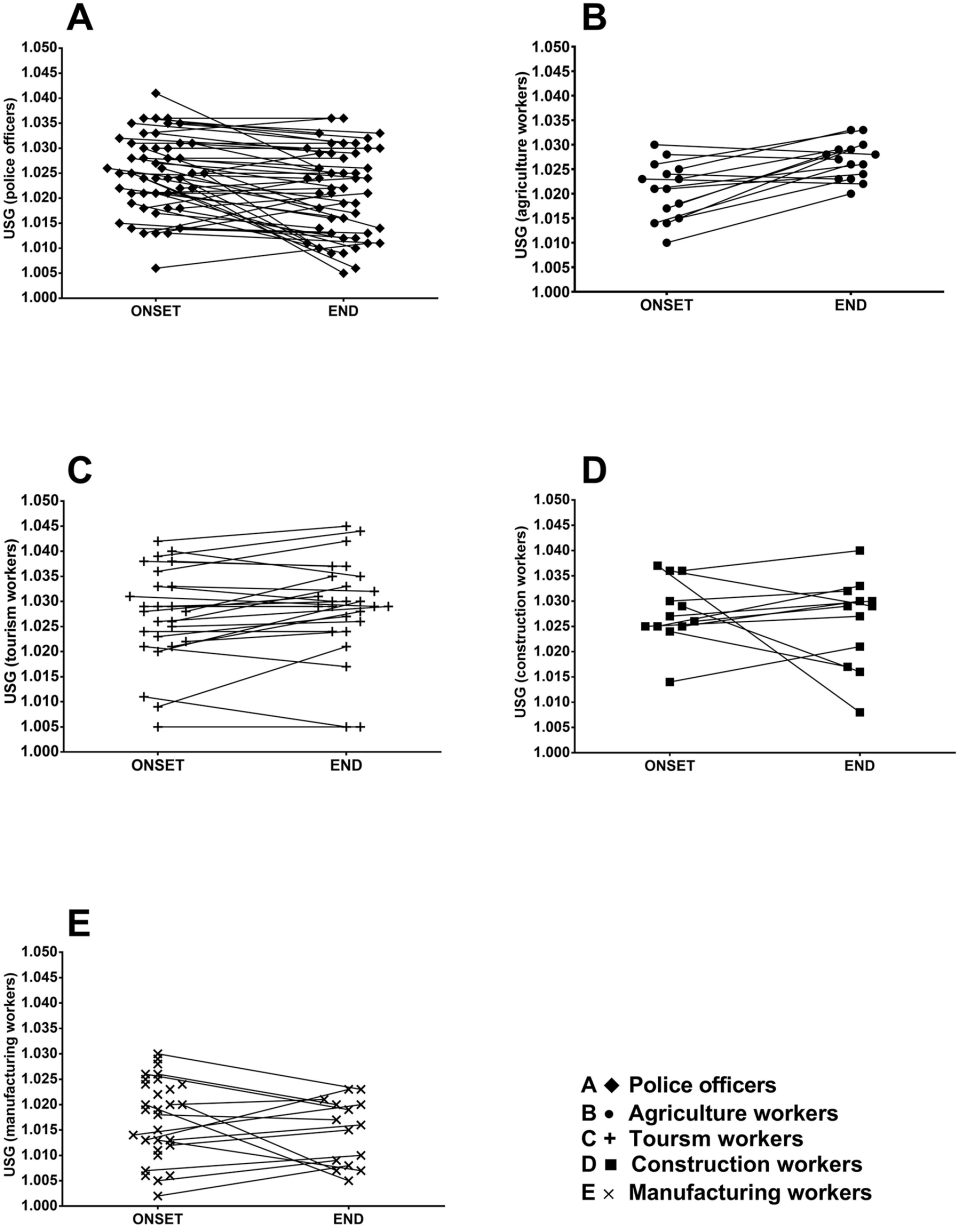
USG values across five different industries in four different European countries. Symbols connected with lines represents participants at the ONSET and END of a work shift. Black diamonds [♦]: Police officers, both inside the office and outdoors, uniform is required. Black dot [●]: Agriculture workers, working outside with no protective clothing requirements. Black crucifix [+]: Tourism workers, inside the office and outdoors. Black square [■]: Construction workers, required to wear protective clothing. Black cross [**X**]: Aluminum extrusion workers, required to wear protective clothing.

The average USG across all industries was 1.023±0.001, at the onset of a work shift and 1.023±0.001 at the end; with 70% (range 44–92% across the five different industries) of the workers initiating work and 69% (range; 14–100%) ending their work shift with a USG level ≥1.020 (see Fig 2).

### Laboratory study

#### T_core_

Values were similar across sessions at BASELINE and elevated by ~1°C, over BASELINE core temperature, in both EUH and DEH MOD conditions (*both P<0*.*001*). T_core_ further increased to 39.3±0.1°C in EUH HYPER and 39.2±0.1°C in DEH HYPER and was significant higher than BASELINE and MOD (*all P<0*.*001)*. No differences between sessions in any conditions was observed (*P<0*.*05*—see Table 1).

**Table 1:** Measures of physiological and psychological strain at baseline, with moderate and hyperthermic levels in the euhydration and dehydration sessions.

	Euhydration (EUH)			Dehydration (DEH)		
	BASELINE	MOD	HYPER	BASELINE	MOD	HYPER
T_core_ (°C)	37.4±0.1	38.5±0.1*	39.3±0.1*#	37.4±0.1	38.5±0.1*	39.2±0.1*#
Body weight (kg)	85.5±4.0	85.7±3.9	85.7±3.8	85.1±3.9	83.4±3.9*#$	83.3±3.9*#$
USG	1.010±0.002	-	1.016±0.003*	1.010±0.003	-	1.020±0.002*
HR (bpm)	82±4	123±6*	131±4*	82±4	117±3*	141±3*#$
TC	0.5±0.0	1.5±0.0*	3.0±0.0*#	0.5±0.0	1.5±0.0*	3.0±0.0*#
TS	1.5±0.0	2.5±0.0*	3.0±0.0*#	1.5±0.0	2.5±0.0*	3.0±0.0*#
RPE	-	12±1*	18±0*#		11±1*	17±0*#

Mean values across sessions and conditions; T_core_ (rectal temperature), body weight, USG (urine specific gravity), HR (heart rate in beats per minute [bpm]), TC (thermal comfort), TS (temperature sensation) and RPE (rate perceived exhaustion) during all trials and conditions. Values are mean±SE.

* significant different from BASELINE,

^#^ significant different from MOD and

^$^ significant different from EUH, (*P<0*.*05*).

#### Hydration (Body weight and USG)

Body weight was significantly lower in DEH MOD and HYPER compared to BASELINE (*both P>0*.*001*) and compared to the EUH session (*all P<0*.*001*). No changes in body weight was observed between BASELINEs and differences between conditions in the EUH session was observed (*P>0*.*05*—see Table 1). USG values in the laboratory were similar at the onset of the experimental days (average of 1.010 and with all individual values below 1.020) and was increased above BASELINE in HYPER to 1.020±0.002, following DEH HYPER and 1.016±0.003, following EUH HYPER (*P<0*.*001 and P = 0*.*032*, *respectively*—see Table 1).

#### Heart rate

HR was higher in MOD compared to BASELINE in both EUH and DEH (*both P<0*.*001*) and higher in HYPER compared to BASELINE and MOD during DEH (*all P<0*.*001)* and higher in HYPER compared to BASELINE in EUH (*P<0*.*001)*. Furthermore, the HR response was augmented in HYPER during the DEH session compared to EUH (*P = 0*.*031*—see Table 1).

#### Thermal comfort rating, temperature sensation rating and rate of perceived exertion

TC and TS followed the same pattern of increase as T_core_, with average rating-scores of ~1.0 in MOD (*both P<0*.*001*) compared to BASELINE. Further, TC and TS was further elevated in HYPER (~3.0, compared to BASELINE and MOD, *both P<0*.*001*). No difference between sessions was observed (*all P>0*.*05*—see Table 1). RPE was elevated in HYPER compared to MOD, but there was no significant differences between sessions (*all P>0*.*05*—see Table 1).

### Performance measures in the simple, complex and combined tasks

#### TARGET_PINCH performance

**Fig 3:**
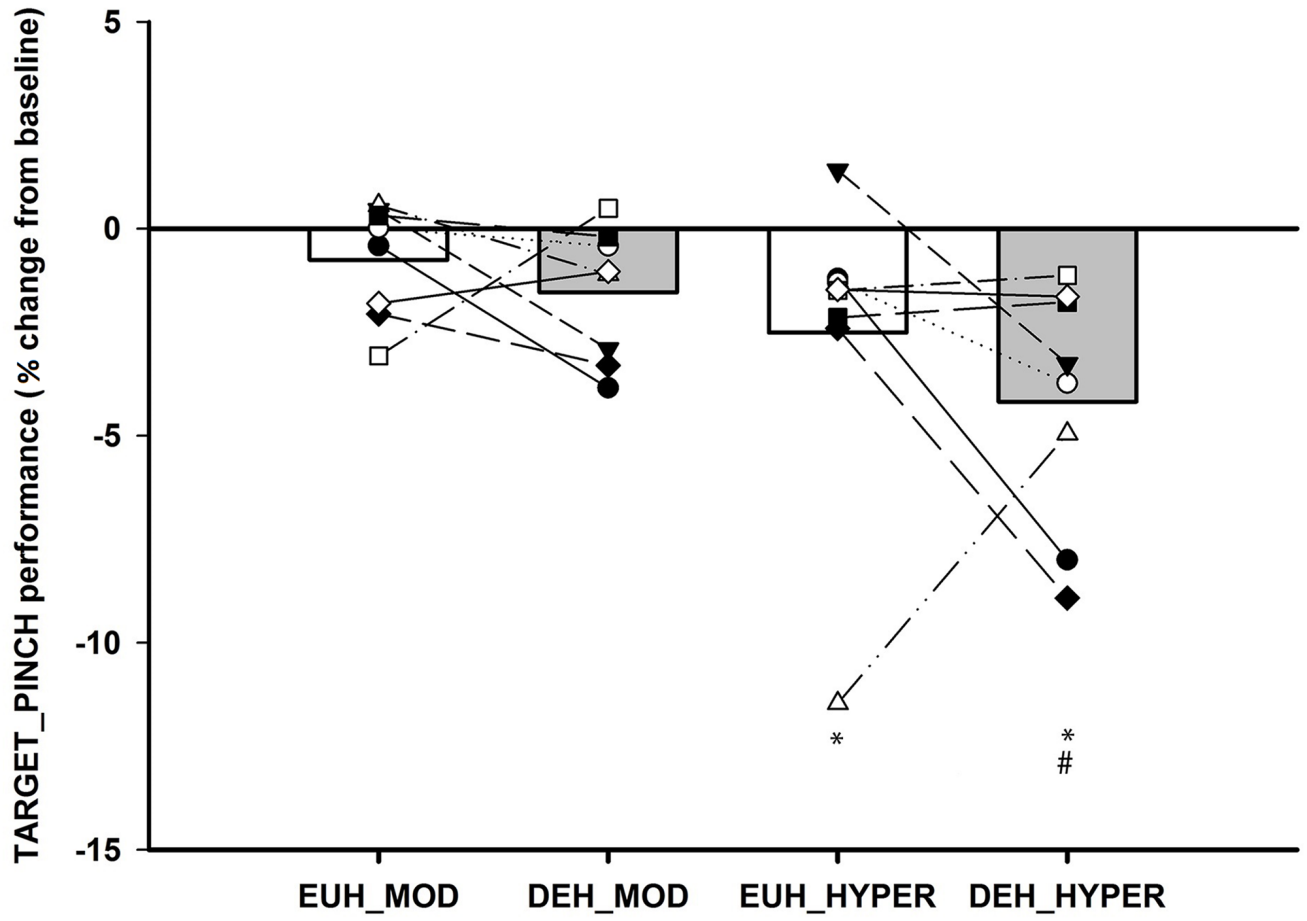
TARGET_PINCH performance score. Individual and mean changes in TARGET_PINCH performance with white bars representing euhydration (EUH: MOD and HYPER) and gray bars the dehydration session (DEH: MOD and HYPER). Values are expressed in percentage change from BASELINE (BASELINE expressed as 0) and individual participant changes in each condition and session. * Significantly different from BASELINE, # Significantly different from MOD *(P<0*.*05)*.

In the DEH session HYPER reduced performance by 4.2±1.0% compared to BASELINE (*P<0*.*001*) and with euhydration HYPER was also associated with a small but significant (*P = 0*.*033)* decrease in performance (*by 2*.*5±1*.*0%*) compared to BASELINE. There were no other differences within the EUH session and no significant differences between sessions (*all P>0*.*05*—see Fig 3 and Table 2).

**Table 2:** Mean performance scores in the four different tasks during euhydration and dehydration with levels of hyperthermia.

	Euhydration (EUH)			Dehydration (DEH)		
	BASELINE	MOD	HYPER	BASELINE	MOD	HYPER
TARGET_PINCH	97.6±0.2	96.9±0.5	95.2±1.3*	97.5±0.6	96.0±0.5	93.4±1.0*#
MATH_TYPE	97.9±0.6	98.3±0.3	96.2±1.1	98.2±0.5	97.2±0.9	94.3±1.4*#
- Errors	4±1	3±1	4±1	3±1	5±1	7±1*#$
MATH_PINCH	88.4±2.4	91.0±1.9	87.0±3.3	90.9±2.6	89.8±2.2	83.1±4.5*#
VMT	77.1±1.2	76.7±1.6	69.2±3.1*#	77.7±1.4	74.7±1.4	65.0±2.9*#

Mean performance scores in percentage of correct answer and time on target (for the VMT task) in conditions (BASELINE, MOD and HYPER) and across sessions (EUH and DEH): TARGET_PINCH, MATH_TYPE and Errors, MATH_PINCH and VMT (visuo-motor tracking), during all sessions and conditions. Values are mean±SE.

* significant difference from BASELINE,

^#^ significant difference from MOD (within session) and

^$^ significant difference compared similar condition in the EUH session (*P<0*.*05*).

#### MATH_TYPE performance

**Fig 4:**
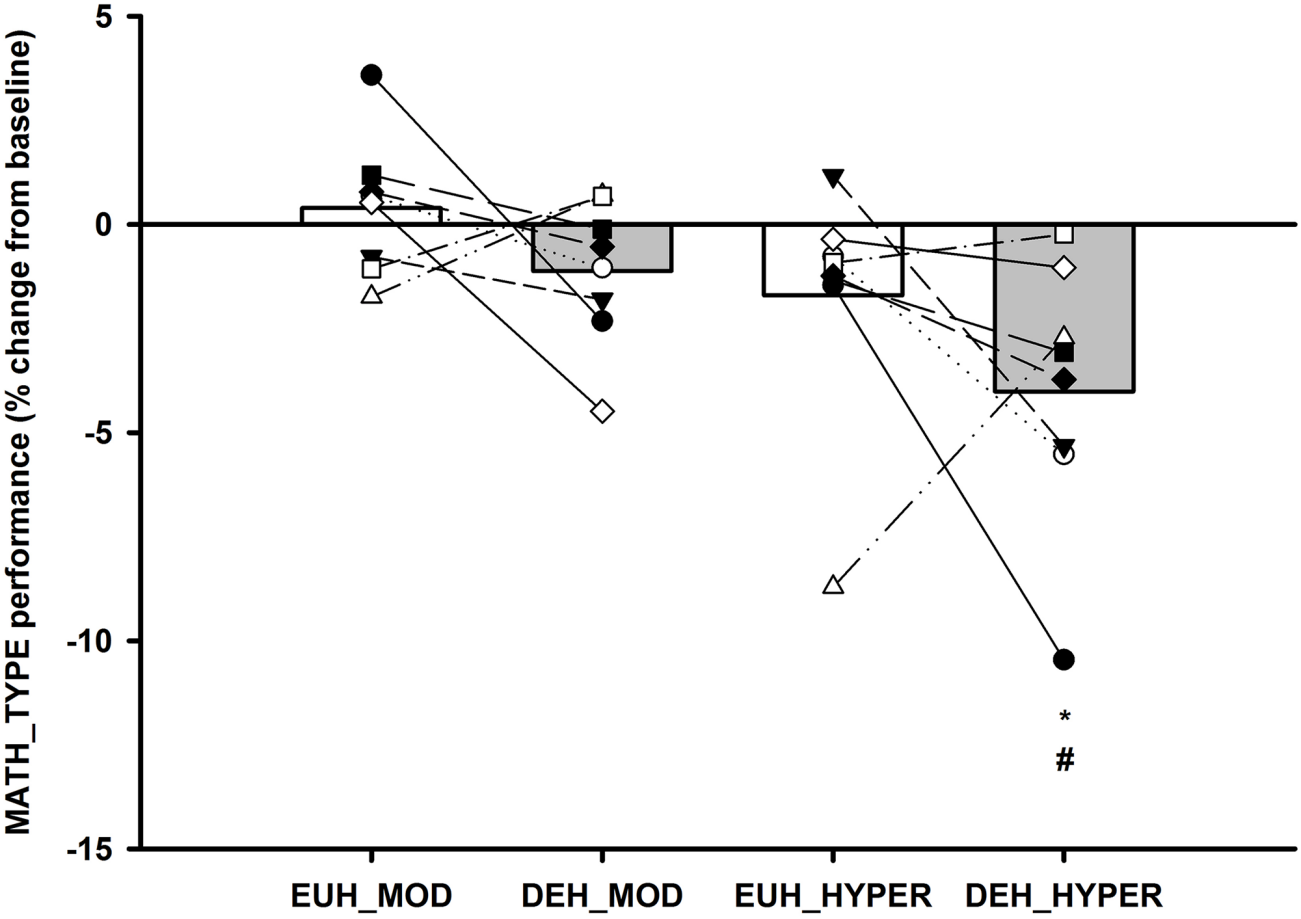
MATH_TYPE performance score. Individual and mean changes in MATH_TYPE performance during euhydration (EUH: MOD and HYPER [White bars]) and dehydration (DEH: MOD and HYPER [Grey bars]). Values are expressed in percentage change from BASELINE (BASELINE expressed as 0) and individual participant changes in each condition and session. * Significantly different from BASELINE and # significantly different from MOD *(P<0*.*05)*.

In the DEH session MATH_TYPE performance was significantly reduced during HYPER compared to BASELINE (*P<0*.*001*) and MOD (*P<0*.*01*). No differences were observed within the EUH session, but there was no significant differences between responses across the two sessions (see Fig 4 and Table 2).

#### MATH_TYPE errors

The number of errors did not change during the EUH session (similar at baseline, MOD and HYPER). In contrast, a significant increase in incorrect answers was observed during the DEH session with more than two fold more mistakes during HYPER compared to BASELINE (*P<0*.*001*) and the occurrence of errors during DEH HYPER was significantly higher compared to EUH HYPER (*P<0*.*05*—see Table 2).

#### MATH_PINCH performance

**Fig 5:**
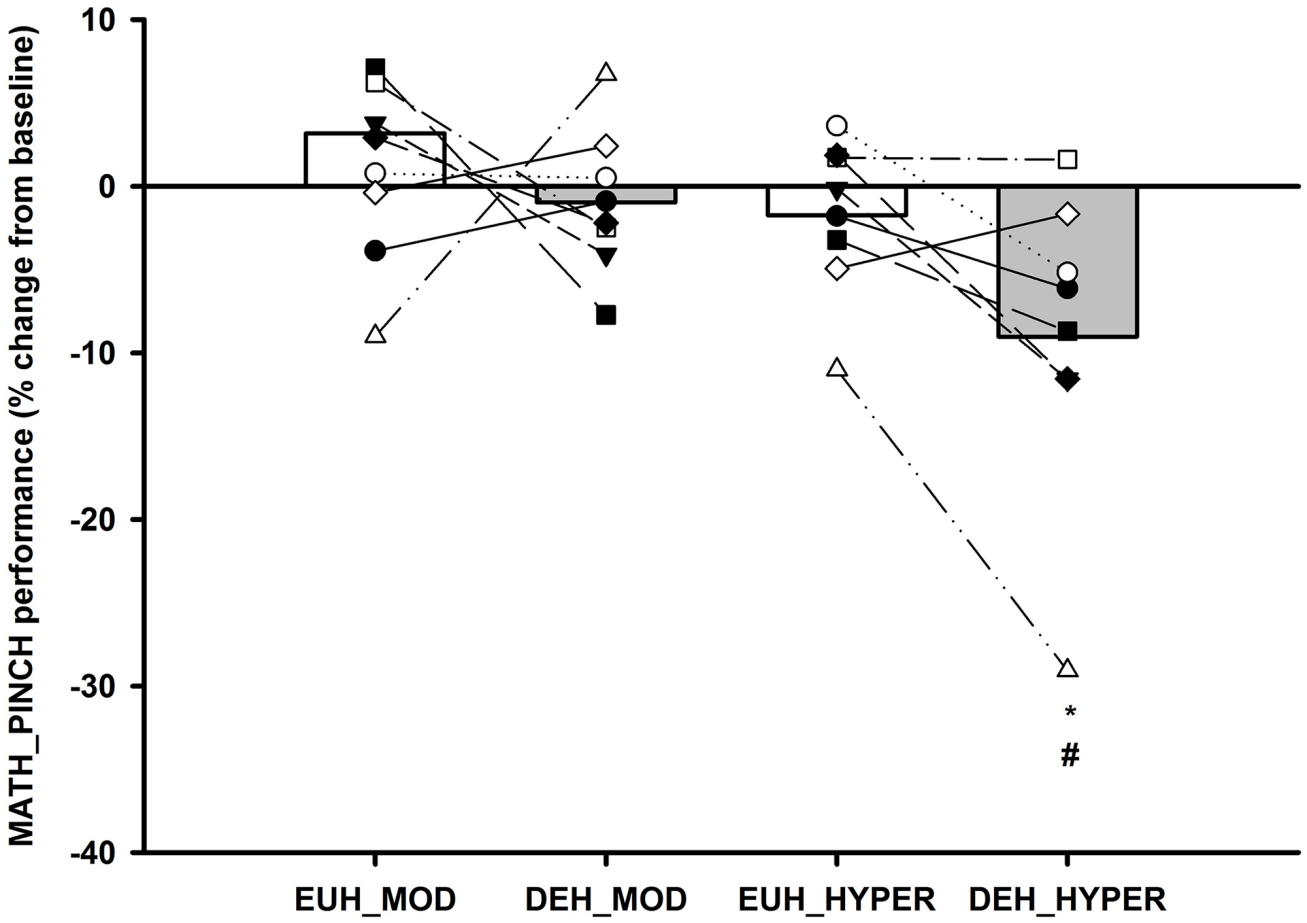
MATH_PINCH performance score. Individual and mean changes in MATH_PINCH performance during euhydration (EUH: MOD and HYPER [White bars]) and dehydration sessions (DEH: MOD and HYPER [Grey bars]). Values are expressed in percentage change from BASELINE and individual participant changes in each condition and session. * Significantly different from BASELINE and # significantly different from MOD *(P<0*.*05)*.

In the DEH session, performance was lowered by 9.0±3.3% in HYPER compared to BASELINE (*P<0*.*001)* and MOD (*P<0*.*01)*. In contrast, during the EUH session no significant differences in performance was observed (*all P>0*.*05)*, but responses across sessions were significantly different (see Fig 5 and Table 2).

#### VMT performance

**Fig 6:**
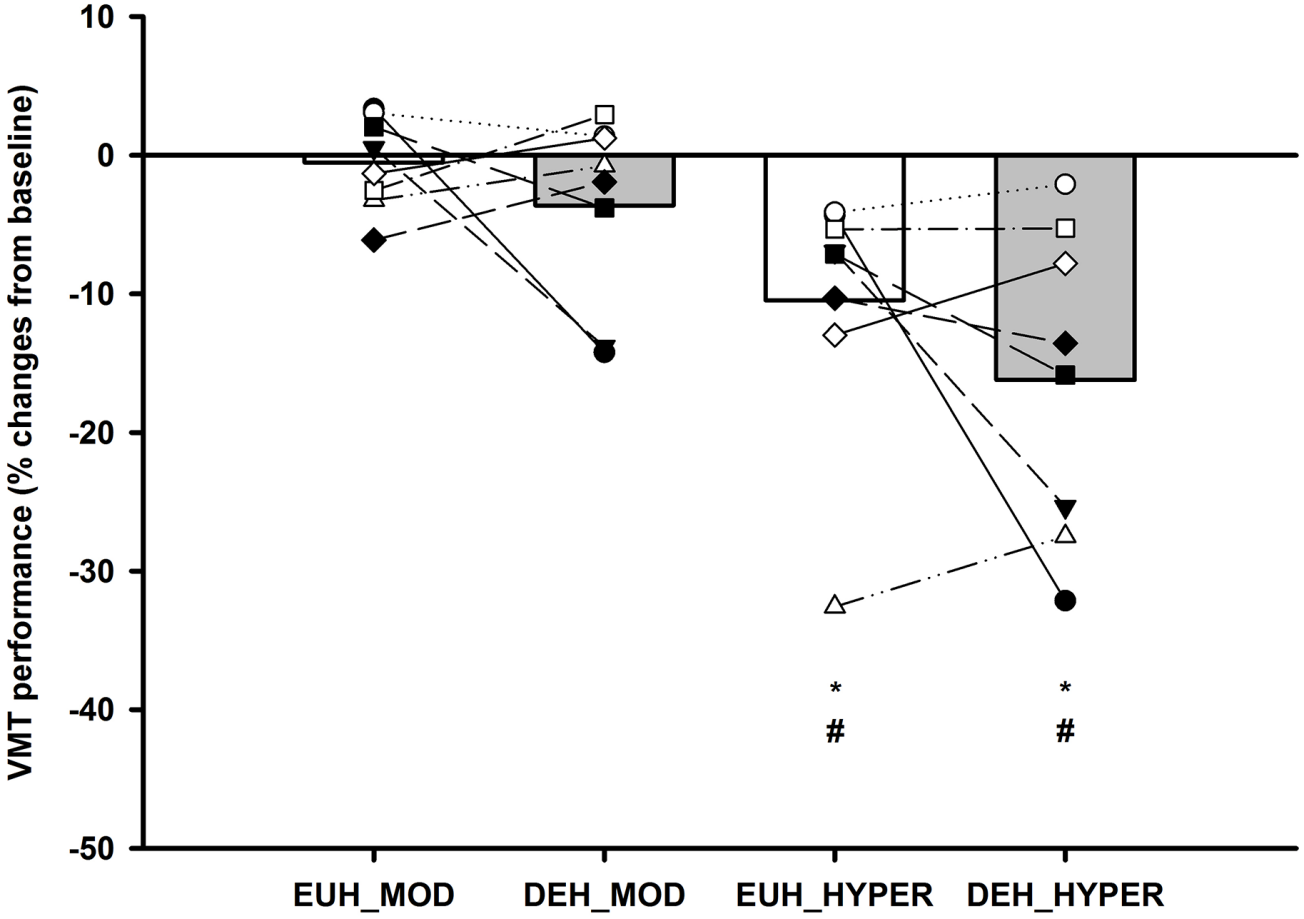
VMT performance score. Performance scores in the visuo-motor tracking (VMT) during euhydration (EUH: MOD and HYPER [White bars]) and dehydration (DEH: MOD and HYPER [Grey bars]). Values are expressed in percentage change from BASELINE (BASELINE expressed as 0) and individual participant changes in each condition and session. * Significantly different from BASELINE and # significantly different from MOD, *(P<0*.*05)*.

HYPER reduced complex motor performance by 10.5±3.3% in the EUH session (*P<0*.*01*) and by 16.2±3.9% (*P<0*.*001*) in the DEH session compared to BASELINE. In both sessions the performance was also impaired from MOD to HYPER (*P<0*.*01)*, but the reduction in performance was not different across sessions (see Fig 6 and Table 2).

## Discussion

The present study demonstrates that two percentage dehydration with corresponding USG values of ~1.020 and encountered in ~70% of all workers across the investigated industries, aggravates the impact of hyperthermia on performance in tasks relying on the interplay between motor and cognitive function and increases the number of errors in a task predominantly relying on cognitive processing. When dehydration was prevented, by matching water intake to sweat loss, the participants tolerated moderate heat stress without any performance decrements. Hyperthermia in the euhydrated state led to decreased performance in the complex VMT task and a minor decrease in TARGET_PINCH task performance, whereas performance in the remaining tasks (simple cognitive and combined motor-cognitive) were not significantly affected. In contrast, when water intake was restricted and the participants became dehydrated, the influence from hyperthermia was aggravated as all task performances were lower in HYPER compared to BASELINE. Whereas, when two-percentage dehydration was combined with moderate heat stress, no task performances were significantly impaired. These findings indicate that dehydration has a moderating effect on the hyperthermia-induced reductions in motor-cognitive performances, wherein dehydration more radically decreases performance at higher levels of hyperthermia.

The performance decrement in the cognitively dominated tasks i.e. MATH_TYPE by 4% and MATH_PINCH by 9%, may seem small. However, when the impact from dehydration is evaluated, it should be considered that the performance was calculated as mean percentage deviation from target/answer. This to allow for quantifying the score during the tasks where the pinch model was used (as it was not possible to compute a perfect”*correct”* result in the pinching task) and to compare between the applied tests. However, the four percentage decrease in MATH_TYPE performance when translated into number of un-correct responses (that may be analyzed and reported for this specific task), corresponded to a doubling of errors and a significantly lower score compared to BASELINE and the EUH HYPER conditions.

For the VMT task, the difference between EUH and DEH was not significant. As demonstrated by our recent study [26] and illustrated in Fig 6, it appears that performance in complex motor tasks is susceptible to influences through hyperthermia compared to simpler tasks and some participants displayed marked negative effects on performance in the euhydration session in the hyperthermic condition with no further effect when dehydration was superimposed. Accordingly, Gaoua and colleagues [43] suggests that complex tasks are affected by hyperthermia to a much larger extent than more stereotypical tasks. Therefore, the performance in choice reaction type of tests may remain unchanged in hyperthermic participants, maybe because some mechanisms within the neural circuitry may outweigh the negative effect of high CNS temperatures or other homeostatic disturbances influencing motor and cognitive function during severe heat stress [25,44–46]. However, when dehydration is superimposed, homeostatic disturbances are aggravated and the combined stress leads to impairments in both combined motor-cognitive and cognitive tasks. The first is in accordance with our hypothesis and indicates that dehydration aggravates the effects of hyperthermia. The present results also suggest that dehydration in combination with moderate increase in core temperature has little or no effect on the ability to perform motor tasks, however a tendency for more mistakes during MATH_TYPE (equals to decreased performance) was observed. This tendency towards lower performance in the applied lab-test, may not be directly translated to lost output/productivity, but our findings may indicate how conditions (hydration in occupations with heat stress) likely will affect the workers capacity. As many occupations rely on both cognition and motor response, this could indeed cause loss of productivity by provoking workers to slow down, make more mistakes etc. that indirectly would cause lowering output e.g. less grapes picked, less aluminum packed etc. and therefore it cannot be excluded that dehydration with moderate heat stress may affect performance in real-life occupations industries. Thus, dehydration may have an even larger impact on performance in cognitive or motor tasks in occupational settings or other daily activities, where people are exposed to high heat stress and fail to prevent hypohydration. In addition, the USG values obtained in the present study across all industries was on average higher compared to the end of the DEH session. More so, when we further distinguish the USG data it revealed that, approximately 23% of all workers had a USG >1.029 corresponding to severely dehydrated/clinically dehydrated [5] and only ~22% of all workers displayed a USG values below 1.015 defined as optimally hydrated [5], at the inset of their work shifts. The prevalence of hypohydration i.e. the fraction of workers with high USG values, in the present study is similar to those reported in some sporting events [47,48] and occupational settings involving moderate to heavy physical activity such as construction [49,50], forest harvesting [51], sugarcane harvesting [6], electrical utility [4] steel and aluminum production and mining [52]. In addition, the WBGT in the above-mentioned studies range from 26–34°C and therefore represent heat stress comparable to the present study. From the present field studies in different European industries and supported by reports from different occupations outside Europe [4–6,49,51,52] it seems that a majority of workers have low hydration status already at the onset of work and that rehydration from day to day may be a bigger issue than failure to drink during the working shift. Aggravation of hydrations status during work shifts was observed only for the agricultural workers, which may relate to the availability of liquids within reach for this group of workers when they perform tasks in the field with limited access to water, whereas average USG values were not worsened for the remaining industries across the working day. Considering that many occupational tasks e.g. handling of industrial machinery, driving, harvesting etc. rely on alertness and the ability to integrate multiple input and react appropriately, therefore, it seems to us that appropriate behavioral changes, regarding fluid intake outside working hours by emphasizing the importance of informing on the consequences of dehydration and the needs for adopting appropriate prevention strategies.

Considering the influence from thermal discomfort or elevated ratings of perceived exertion, we did not observe across session differences in those parameters (similar elevation in RPE, TS and TC from BASELINE to MOD and HYPER in the euhydration and dehydration sessions) and the observed dehydration-induced impairments in cognitive and motor performances are not directly related to differences in those parameters. The performance impairments caused by dehydration can therefore not be directly ascribed to alterations in the participant’s perception of thermal discomfort or elevated exertion in the dehydration trial. However, dehydration would likely aggravate all of the above factors in real-life setting as hypohydration impairs thermoregulatory function during exposure to elevated environmental temperatures [53].

The physiological mechanisms of importance for this interaction remain to be elucidated, but could involve increases in cardiovascular and thermal stress with associated aggravation of thermal discomfort when dehydration is superimposed. The higher heart rate associated with dehydration (141 vs. 131 bpm) equivalent to 7% increase in dehydration HYPER compared to euhydration HYPER, signifies an overall elevation of cardiovascular stress. Although it is below 65% of the participants’ age predicted heart rate reserve, it cannot be excluded that it evoked or influenced the overall sensation of stress. In accordance with Cheuvront and colleagues [15] the decrease in exercise performance is attributed to the increased thermal strain on the circulatory system and perceived exhaustion, which contributes to behavioral changes. Changes in cerebral blood flow (CBF) would clearly be a cardiovascular factor that directly could influence cognitive function. Previous studies have shown that CBF decreases in hyperthermic participants [54] with the decline accelerated when dehydration is not prevented during the heat exposure [24]. However, the lower CBF observed with hyperthermia or combined dehydration and hyperthermia is counteracted by increased oxygen and glucose extraction across the brain [23]. Accordingly, the cerebral metabolic rates for oxygen and glucose are maintained or even slightly elevated in hyperthermic participants (see [54] for discussion of the Q_10_ effect on the cerebral metabolic rate) and alterations in cerebral metabolic function cannot directly explain the fatigue observed during prolonged exercise in the heat with or without dehydration [23,24,54]. Whether, it may influence the ability to increase regional CBF and supply the activated brain areas with adequate oxygen and substrates, during the more complex cognitive and motor tasks applied in the present study remains unknown. It could indeed be of importance since complex tasks are associated with higher regional CBF during activation compared to simpler tasks [25,55]. In addition increased brain area activation observed with dehydration or hyperthermia [38,56] and changes relating to motor response e.g. changes in the neural circuit (reduction in H-reflex amplitude, lowering of EMG signal, adjustment in force frequency etc.) observed in the heat [21,57,58]. These mechanisms are likely to contribute to the decreased performance in both laboratory testing and in real world scenarios, when superimposition of dehydration is present, although this has yet to be elucidated.

The results from the present study let us conclude that dehydration at levels commonly observed in occupational settings in a variety of industries aggravates the impact of hyperthermia on performance in tasks relying on the interplay between motor and cognitive functions. In accordance with our first hypothesis, the largest performance decrements provoked by combined dehydration and hyperthermia were observed for the combined motor-cognitive tasks. Although, there was no significant difference between sessions, it was the complex motor task that displayed the largest decrease of all tasks when subjects were exposed for high heat stress in the dehydrated session. In relation to our second hypothesis, the prevalence of dehydration across the evaluated industries was high and dehydration is apparently still a prevailing problem in occupations with heat stress. These findings let us suggest that prevention plans with implementation of suitable and more effective hydration and rehydration strategies are warranted at work places to minimize the negative effects of dehydration on workers performance when they are exposed to occupational heat stress.

## Supporting information

S1 Data Data. (XLS)
